# Stabilization of Zinc in Agricultural Soil Originated from Commercial Organic Fertilizer by Natural Zeolite

**DOI:** 10.3390/ijerph19031210

**Published:** 2022-01-22

**Authors:** Lijuan Sun, Shuangxi Li, Peiyun Gong, Ke Song, Hong Zhang, Yafei Sun, Qin Qin, Bin Zhou, Yong Xue

**Affiliations:** 1 ECO—Environment Protection Research Institute, Shanghai Academy of Agricultural Sciences, Shanghai 201403, China; sunlijuan@saas.sh.cn (L.S.); lishuangxi@saas.sh.cn (S.L.); gongpy233@126.com (P.G.); songke@saas.sh.cn (K.S.); zhanghong1878536@163.com (H.Z.); sunsunsun_cool@126.com (Y.S.); qinqin@saas.sh.cn (Q.Q.); zhoubean@saas.sh.cn (B.Z.); 2 Shanghai Environmental Protection Monitoring Station of Agriculture, Shanghai 201403, China; 3 Shanghai Key Laboratory of Protected Horticultural Technology, Shanghai Academy of Agricultural Sciences, Shanghai 201403, China; 4 Shanghai Rightway Environmental Protection Technology Co., Ltd., Shanghai 200131, China

**Keywords:** organic fertilizer, zinc, zeolite, Chinese cabbage

## Abstract

Exploring ways to reduce the risk of heavy metal pollution by organic fertilizer application is of vital importance. In the present study, by conducting a pot experiment, natural zeolite was applied together with pig manure based organic fertilizer to agricultural soil in order to test its possibility of reducing the risk of heavy metals originating from pig manure. The results showed that a low rate of organic fertilizer (10%) application increased the biomass of Chinese cabbage (by 57.2%), while a high rate of organic fertilizer (30%) decreased the biomass of Chinese cabbage (by 46.16%), and meanwhile a 3% zeolite addition increased the biomass of Chinese cabbage which was treated with 30% organic fertilizer. The organic fertilizer addition decreased soil pH and increased soil CEC, while zeolite addition increased soil pH and decreased the soil organic matter content. The concentration of Zn in Chinese cabbage shoots increased with the organic fertilizer addition from 4.46% to 48.27%, while the addition of 1% and 3% zeolite significantly decreased Zn in Chinese cabbage shoots by 15.53% and 14.08%, respectively. The concentration of DPTA-extractable and DGT-extractable Zn of soil was increased by organic fertilizer application, whereas zeolite addition decreased the concentration of DPTA-extractable and DGT-extractable Zn in soil treated with organic fertilizer. Our present study suggests that natural zeolite application could be a promising method to reduce the risk of heavy metals originating from organic fertilizers.

## 1. Introduction

As the biggest pork-producing country in the world, China produces more than half of the total world’s pork [1]. It is estimated that there are 660 million pigs in China [2]. Intensive pig production for human food is always associated with a massive production of pig manure (PM). Pig manure is traditionally applied to agricultural soils with a double objective, as a fertilizer and to recycle waste material [3]. Application of PM increases soil nutrient content, pH and available water, and decreases the bioavailability of heavy metals due to it being rich in nitrogen, phosphorus and organic matter [4]. The application of PM to agricultural fields is considered to be an environmentally-friendly method of waste disposal.

However, the contents of copper (Cu) and zinc (Zn) in pig manure are relatively high, as Cu and Zn are usually added to pig feeds to maintain various physiological processes and prevent animal health disorders and are excreted through feces and urine [5]. A strong correlation was observed between the total Cu and Zn contents in soil and the application rate of PM; continuous application of PM in agricultural soil will reach the ceiling concentration limits of Cu and Zn in soil only in 9 years [6]. In addition, the concentration of Cu and Zn in crops was strongly correlated with the extractable Cu and Zn in soil [6,7]. Therefore, continuous application of pig manure containing relatively great concentrations of Cu and Zn raises environmental concern for the toxicity caused by high levels of those metals to organisms.

The toxicity of heavy metals to terrestrial organisms largely depends on its bioavailability in soil. Soil chemical properties such as pH, redox potential (Eh), content of organic matter, cation exchange capacity and content of clays, iron (Fe), and manganese (Mn) oxides are the main environmental factors affecting Cu bioavailability [8]. Amending soil with proper amendments to change the bioavailability of heavy metals is not a new concept and has been practiced for a long time in the remediation of agricultural soil. Zeolite is a naturally found mineral of the aluminosilicates group. Zeolite is widely applied to heavy metal polluted soils to immobilize and reduce the uptake of heavy metals by plants due to its advantageous features such as a large surface area, negative charge and high cation exchange capacity (CEC) values [9,10]. Zeolite has been shown to have a selective stabilization effect on soil Cd [11]. The application of zeolite increased soil pH at 0.21–0.72 and reduced the available Cd by 18.5–31.0% [12]. The immobilization of zeolite on Zn in pig manure applied in soil has not yet been reported.

By conducting a pot experiment, the aim of the present study was to: (1) evaluate the effect of different rates of pig manure based organic fertilizer application on Zn content in plants; (2) investigate the effect of zeolite on soil property and Zn bioavailability in soil and accumulation in plants.

## 2. Materials and Methods

### 2.1. Experimental Soil, Organic Fertilizer and Natural Zeolite

Vegetable soil was collected from Zhuanghang comprehensive test station of Shanghai Academy of Agricultural Sciences, Shanghai. The basic properties were pH 6.61, organic matter content 18.9 g/kg, total nitrogen (N) content 1.09 g/kg, olsen potassium content 85.98 mg/kg, total phosphorus (P) 32.58 mg/kg, total Cu content 43.01 mg/kg and total Zn content 113.14 mg/kg. After removal of visible rocks, roots and fresh litters, the soil was air-dried, ground to particle size of less than 2 mm. Organic fertilizer was supplied by Shanghai Juyuan Organic Fertilizer Limited Company. The organic fertilizer was mainly fermented by pig manure. The basic properties of organic fertilizer were: water content 9.62%, organic matter content 77.13%, total N 4.07%, total P (P_2_O_5_) 5.88%, total K (K_2_O) 2.78%, pH 8.43, total Cu 295.32 mg/kg, total Zn 1696.09 mg/kg. Natural zeolite was supplied by Shanghai Shengnong International Trade Company. The zeolite was collected from one of the oldest commercial deposits in New South Wales, Australia. The zeolite consisted of 85% clinoptiloite and 15% geolyte. It was a kind of natural mineral, without any addition. The substance composition was silicon dioxide (SiO_2_) 71.81%, aluminum oxide (Al_2_O_3_) 12.1%, ferric oxide (Fe_2_O_3_) 1.14%, sodium oxide (Na_2_O) 2.33%, potassium oxide (K_2_O) 0.9%, calcium oxide (CaO) 2.6%, magnesium oxide (MgO) 0.65%, titanium dioxide (TiO_2_) 0.22%, manganese oxide (MnO) 0.03%, strontium oxide (SrO) 0.22%, total Cu 19 mg/kg, total Zn 33 mg/kg. The water content was less than 5% and CEC value was 147 meq/100 g. The particle size of the zeolite was 1~3 mm.

### 2.2. Experimental Design

Using a pot experiment (23 cm in diameter), seven treatments replicated four times each were sets. Organic fertilizer (fermented from pig manure) with three rates (0, 10% and 30%) together with three rates of zeolite (0, 1% and 3%) were mixed with 1.5 kg air-dried soil at the beginning of the experiment. After mixing thoroughly, all the pots were placed in a glasshouse under controlled condition (70% relative humidity, day/night duration 16/8 h, and day/night temperature 25/20 °C). Distilled water was added every three days to keep about 70% soil water capacity for two months to age the soil. After aged for two months, Chinese cabbage (*Brassica chinensis* L.) seeds were sowed in the surface of soil. About 15 days later, all 28 pots were thrown down to two plants in the center of the pots. Soils and plants sample were collected after sowed for 40 days.

### 2.3. Soil and Plant Analysis

All the soil samples were freeze-dried and stored in drier for further analysis. Plant samples were divided into two portions. One was stored in ultra-cold storage freezer (−80 °C) for further analysis and the other portion was firstly dried in an oven under 105 °C for 30 min and then dried under 65 °C to the constant weight and later on stored in drier for further analysis.

#### 2.3.1. Soil pH and CEC Value

Soil pH was measured by a pH meter (Mettler Toledo, Zurich Switzerland), at a soil-to-water ratio of 1:2.5. The CEC value was measured by the extraction method, using sodium acetate and ammonium acetate.

#### 2.3.2. Zn Concentration in Plants

Plant samples were digested with concentrated nitric acid—30% hydrogen peroxide (H_2_O_2_)-hydrofluoric acid (HF) in the microwave digestion apparatus (CEM Mars One, Matthews, NC, USA). Total amount of Zn in the digestion solution was determined by ICP-MS (7900, Agilent Technology, Santa Clara, CA, USA).

#### 2.3.3. Chemical Speciation of Zn in Soil

Phytoavailable Zn in soil was extracted by diethylenetriaminepentaacetic acid mixture (DTPA (0.005 M DTPA, 0.1 M triethanolamine, and 0.01 M calcium chloride)) at pH 7.3 for 2 h with a soil/solution ratio of 1:5 (*w*/*v*) [13]. After centrifugation for 10 min at 12,000× *g*, the supernatant liquid was filtered through quantitative filter papers and element concentrations were determined by flame atomic absorption spectroscopy (FAAS). Zinc speciation of soil was operationally determined by a selective sequential extraction [14]; the Zn fractions in soil were defined as exchangeable, bound to carbonates, bound to iron and manganese oxides, and bound to organic matter and residual Zn. Soil sample (1.00 g) was freeze-dried, sieved (<150 μm), and placed in 50 mL polypropylene centrifuge tubes before the extraction procedures. Zinc concentration of each extract was determined by ICP-MS.

#### 2.3.4. Diffusive Gradients in Thin Films (DGT) Extractable Zn in Soil

Diffusive gradients in thin films technique is based on Fick’s first diffusion law, which is widely used to in situ measure the content of the biological effective metals in environmental media. The DGT device consists of a diffusive layer with polyacrylamide and DGT crosslinker overlying a restricted gel layer containing Chelex-100 resin, a dialysis membrane, as well as plastics molding. The cylindrical DGT devices (Chelex DGT, Nanjing, China) used in the present study were purchased from Easy Sensor (Nanjing, China). The freeze-dried soil samples were analyzed by DGT according to the method described in the Supplementary Material (Figure S1).

### 2.4. Data Analysis

Analysis of one-way ANOVA on plant biomass and concentrations of elements were performed using Windows-based SPPS 16.0 (SPSS Inc., Chicago, IL, USA). Data are represented as mean value ± standard deviation (SD) (four treatments) in all figures. The significance levels (*p* < 0.05) between different treatments were determined by the Fisher least significant difference (LSD) test.

## 3. Result

### 3.1. The Growth of Chinese Cabbage

**Figure 1:**
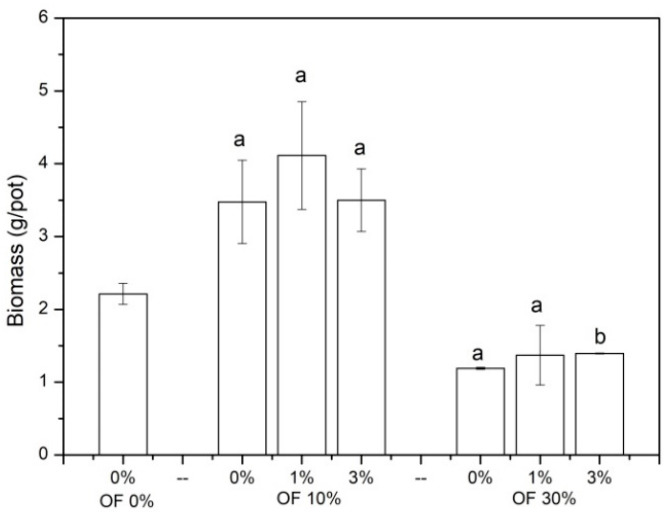
The biomass of Chinese cabbage in different treatments. (OF: organic fertilizer. Values were mean ± standard deviation. Values followed by different letters within a column indicate significant difference (*p* < 0.05) at LSD test).

The biomass of Chinese cabbage is shown in Figure 1. For all of the treatments without zeolite addition, when compared with the control without organic fertilizer application, the application of 10% organic fertilizer increased the biomass of Chinese cabbage by 57.2%, while the application of 30% organic fertilizer decreased the biomass of Chinese cabbage by 46.16%. A trend of increase for Chinese cabbage biomass was found with the addition of zeolite. In treatments with 30% organic fertilizer, the biomass of Chinese cabbage was significantly increased by 3% zeolite addition (*p* < 0.05).

### 3.2. Soil Properties

**Figure 2:**
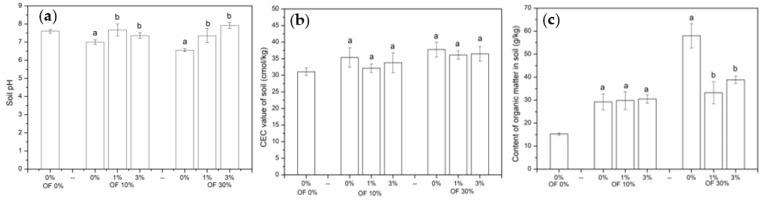
The pH (**a**), CEC (**b**) and organic matter content (**c**) value of soil in different treatments. (OF: organic fertilizer. Values were mean ± standard deviation. Values followed by different letters within a column indicate significant difference (*p* < 0.05) at LSD test).

The pH, CEC value and organic matter of soil are shown in Figure 2. The soil pH was slightly decreased by the application of organic fertilizer. In soil treated with 10% and 30% organic fertilizer, the soil pH value was significantly (*p* < 0.05) increased by zeolite addition (Figure 2a). In soil treated with 10% organic fertilizer, the pH value increased (*p* < 0.05) by 0.67 and 0.30 units by 1% and 3% zeolite addition, respectively. In soil treated with 30% organic fertilizer, the pH value increased (*p* < 0.05) by 0.80 and 1.37 units by 1% and 3% zeolite application, respectively. The CEC value of soil ranged from 31.05 to 37.76 cmol/kg (Figure 2b) and organic matter application increased soil CEC value by 13.75% to 21.31%. The soil organic matter ranged from 15.28 to 57.97 g/kg. The organic matter content of soil with 10% and 30% organic fertilizer addition was 1.91 and 3.79 times that of the soil without control (Figure 2c). In soil treated with 10% organic matter, zeolite addition showed no significant difference on soil organic matter. In soil treated with 30% organic matter, the addition of 1% and 3% zeolite decreased the content of soil organic matter by 42.71% and 33.02%, respectively.

### 3.3. Zn Concentration in Chinese Cabbage Shoots

**Figure 3:**
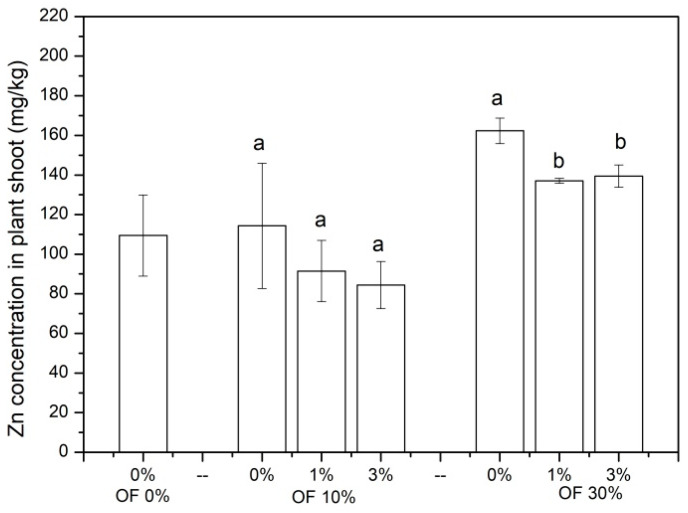
The concentration of Zn in plant shoot in different treatments. (OF: organic fertilizer. Values were mean ± standard deviation. Values followed by different letters within a column indicate significant difference (*p* < 0.05) at LSD test).

The concentration of Zn in Chinese cabbage is shown in Figure 3. The concentration of Zn in Chinese cabbage shoots ranged from 84.41 to 162.28 mg/kg, and it was increased by organic fertilizer application from 4.46% to 48.27%. In soil treated with 30% organic fertilizer, the addition of 1% and 3% zeolite significantly (*p* < 0.05) decreased Zn in Chinese cabbage shoots by 15.53% and 14.08%, respectively. A trend of decrease for Zn concentration in Chinese cabbage shoots by zeolite addition was also found in soil treated with 10% organic fertilizer, although no statistical difference was found.

### 3.4. The Concentration of Zn in the Soil Extracted by Single-Step Chemical Extraction and DGT

**Figure 4:**
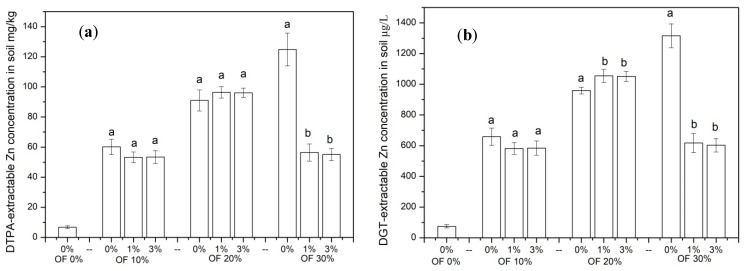
The concentration of DTPA-extractable Zn (**a**) and DGT-extractable Zn (**b**) in soil of different treatments. (OF: organic fertilizer. Values were mean ± standard deviation. Values followed by different letters within a column indicate significant difference (*p* < 0.05) at LSD test).

DTPA-extractable Zn, which represented the bioavailability of Zn in soil, is shown in Figure 4a. In soil treated without organic fertilizer, the concentration of DTPA-extractable Zn was only 6.8 mg/kg. However, the DTPA-extractable Zn in soil increased significantly with organic fertilizer application. In soil treated with 30% organic fertilizer, the concentration of DTPA-extractable Zn reached 124.9 mg/kg. In soil treated with 10% organic fertilizer, no significant difference was found among different zeolite treatments. However, in soil treated with 30% organic fertilizer, the addition of 1% and 3% zeolite significantly (*p <* 0.05) decreased the concentration of DTPA-extractable Zn by 54.8% and 55.9%. The concentration of Zn extracted by DGT is shown in Figure 4b. In soil treated without organic fertilizer, the concentration of DGT-extractable Zn was only 74.2 μg/L. In soil treated with 30% organic fertilizer, the application of 1% and 3% zeolite significantly (*p <* 0.05) decreased the concentration of DGT-extractable Zn by 53.1% and 54.2%.

### 3.5. Chemical Speciation of Zn in Soil

**Figure 5:**
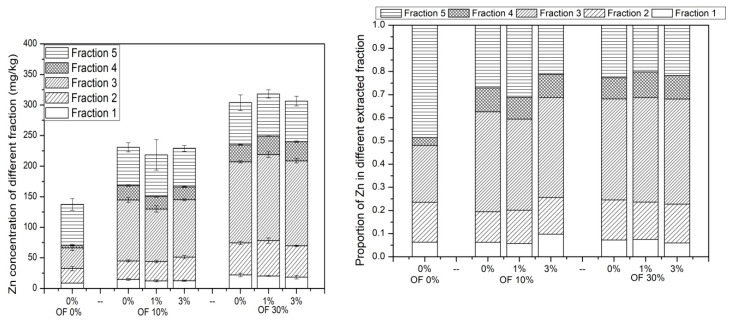
Concentration and percentage of Zn present in each fraction of soil subjected to different treatments. (OF: organic fertilizer. Fraction 1: exchangeable, fraction 2: bound to carbonates, fraction 3: bound to iron and manganese oxides, fraction 4: bound to organic matter, fraction 5: residual).

The concentration and proportion of the five Zn fractions are shown in Figure 5. The concentrations of fraction 4 (bound to organic matter), fraction 3 (bound to iron and manganese oxides), fraction 2 (bound to carbonates) and fraction 1 (exchangeable) of Zn in soil were increased by organic fertilizer application. However, organic fertilizer application showed no significant influence on the concentration of fraction 5 (residual) of Zn in soil. In soil treated with 10% organic fertilizer, the application of zeolite decreased the concentration of Zn in fraction 1, fraction 3 and fraction 4; however, the concentration of Zn in fraction 2 increased. In soil treated with 30% organic fertilizer, the addition of zeolite decreased the concentration of Zn in fraction 1, while the concentration of Zn in fraction 2, fraction 3 and fraction 4 increased. The concentration of Zn in fraction 5 showed no significant difference among all of the seven treatments.

## 4. Discussion

The application of organic fertilizer increased the yield and quality of crops when used appropriately by providing the essential macro- and micro-nutrients, as well as an array of plant growth-promoting substances [15]. Animal manure is abundant in nutrients, promoting plants’ vegetative growth and ensuring stable and robust production. The yields of horticultural crops such as maize [16], potato [17] and tomato [18] have been reported to be improved by animal manure application. By conducting an 8-year field experiment, Cai et al. [16] reported that the application of pig manure increased soil total N, available N, total P and available P, leading to an increase in crop yield. Zhang et al. also found that the application of composting pig manure improved rice biomass and yield [19]. In the present study, the application of 10% pig manure based organic fertilizer increased the biomass of Chinese cabbage, while the application of 30% pig manure based organic fertilizer decreased the biomass of Chinese cabbage. Excessive organic fertilizer application may lead to decrease in crop yield due to the dilution impact of excess organic matter with high nitrogen supply [20]. The result of the present study could be explained by the excessive nitrogen supply and dilution of organic matter by excessive pig manure application. High nitrogen concentration decreased calcium in leaves of tomato crops and increased tomato fruit blossom-end rot in Guyana [21].

Organic residues from green and animal manure can influence the properties of soil and improve soil fertility by supplying nutrients for crop production [21,22]. The effect of animal manure on soil pH may persist over several years [23]. In the present study, the pH of soil was found to slightly decrease due to the application of organic fertilizer. The mineralization of organic matter in soil may lead to the release of acidic compounds. In addition, microbial metabolic activity in soil enhanced the release of acid gas such carbon dioxide which retained in soil, leading to the decrease of soil pH [24]. Guo et al. also reported that the pH value (7.00) of soil with organic matter addition was slightly lower than that of soil without organic matter addition (7.14) [25]. It has been suggested that pH and CEC value are the most two important factors that control the effect of zeolite on heavy metal bioavailability in soil. The addition of zeolite increased both soil pH and CEC value in soil, but Querol et al. considered that the increasing of soil pH caused by zeolite addition was the main reason for the decrease of Pb bioavailability in soil [26]. In our present study, the addition of zeolite showed no significantly different influence on soil CEC value. The significant increase of pH value caused by zeolite may be one of the reasons for the decrease of Zn bioavailability.

Zeolite has been developed to serve as a soil conditioner as it is a crystalline, hydrated aluminosilicate of alkali and alkaline earth cation having an infinite, open, three-dimensional structure [27]. In aqueous solutions, the adsorption of zeolite on heavy metals such as Pb^2+^, Cu^2+^, Cd^2+^ and Ni^2+^ has been proven and well-studied [28,29]. Forming oxides, complexes and metal-carbonate precipitates and regulating soil pH value are the main mechanisms of zeolite that decrease metal solubility in soil [26,30,31]. Peng et al. found that the addition of 2.5% zeolite reduced the mobile fraction (including soluble, exchangeable, and carbonate-bound fraction) Pb, Cu and Cd in amendments, thus reducing the concentration of metal in leachates of soil [32]. In the present study, the application of zeolite significantly decreased the concentration of Zn in fraction 1, DTPA-extractable Zn and DGT-extractable Zn in soil treated with organic fertilizer. Stabilization of the heavy metals in organic fertilizer by zeolite addition ensured an environmentally-friendly method to use organic matter in soil and made the organic fertilizer treatment operational and cost-effective.

## 5. Conclusions

An appropriate rate of pig manure based organic fertilizer application promoted the growth of Chinese cabbage, while a high rate (30%) of organic fertilizer application showed toxicity to Chinese cabbage, and the toxicity could be alleviated by zeolite addition. Organic fertilizer addition decreased the soil pH and increased CEC as well as soil organic matter content, while zeolite addition increased the soil pH and decreased the soil organic matter content. The application of organic fertilizer increased the concentration of Zn in Chinese cabbage shoots, whereas the addition of zeolite significantly decreased Zn in Chinese cabbage shoots. The concentration of DPTA-extractable and DGT-extractable Zn of soil was increased by organic fertilizer addition, while zeolite decreased the concentration of DPTA- and DGT-extractable Zn in soil treated with organic fertilizer. Our present study suggests that natural zeolite application could be a promising way to reduce the environmental risk of heavy metals originating from organic fertilizer.

## Data Availability

Not applicable.

## Supplementary Materials

The following supporting information can be downloaded at: https://www.mdpi.com/article/10.3390/ijerph19031210/s1, Figure S1: Pictures of Chinese cabbage treated with different rate of organic fertilizer.
