# Supplementary material for: Stabilization of Zinc in Agricultural Soil Originated from Commercial Organic Fertilizer by Natural Zeolite
Source: Int J Environ Res Public Health. 2022 Jan 22;19(3):1210. doi: 10.3390/ijerph19031210 (PMC8835060; doi:10.3390/ijerph19031210)
Supplement: Supplementary file 1 [file ijerph-19-01210-s001.zip › ijerph-1547421-supplementary.pdf]

Stabilization of Zinc in Agricultural Soil Originated from Commercial Organic Fertilizer by Natural Zeolite

Lijuan Sun [1,2,3,4,†], Shuangxi Li [1,2,3,†], Peiyun Gong [1,2,3], Ke Song [1,2,3], Hong Zhang [1,2,3], Yafei Sun [1,2,3], Qin Qin [1,2,3], Bin Zhou [1,2,3] and Yong Xue [1,2,3,*]

Zn in Soil Extracted DGT

A total amount of 10 g soil samples were placed in clean beaker, added with Milli-Q water to obtain 70% maximum field water holding rate, mixed thoroughly with a glass bar and equilibrated at 25 ± 1 °C for 48 h. A plastic wrap was kept on the beaker to avoid the evaporation of water. About 3 g soil was then put into the DGT units by a clean plastic spoon and shaken gently on the desk to make soil fully exposed to the filter membrane. DGT units with soil was maintained for 24 h at 25 ± 1 °C, then were removed from the soil and washed with Milli-Q water. The resin gel was immersed in 1 mol/L HNO_3 for 24 h and the metal contents were measured by ICP-MS. The effective concentration of DGT (C_{DGT}) was calculated according to the following Equation (1) [1,2].

$$C_{DGT} = \frac{C_e(V_e + V_g) \cdot \triangle g}{D \cdot A \cdot t \cdot f_e} \quad (1)$$

where C_e is the concentration of metals in the elution solution, V_e is the volume of HNO_3 added to the resin gel (1.8 mL), V_g is the volume of the resin gel (0.2 mL), Δg is the thickness of diffusive layer (cm); D is the diffusion coefficient of metallic element in the diffusive layer ($cm^2 \cdot s^{-1}$); A is the area of DGT exposure window (3.14 cm^2); and t is the DGT deployment time (s), and f_e is the elution factor for the metals (0.938).

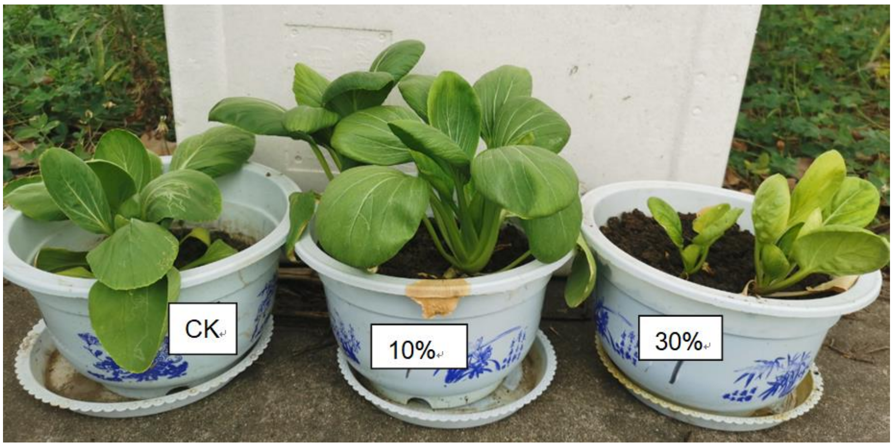

Figure S1. Pictures of Chinese cabbage treated with different rate of organic fertilizer.

Reference

1. Zhang, H.; Davison, W. Performance-Characteristics of Diffusion Gradients in Thin-Films for the In-Situ Measurement of Trace-Metals in Aqueous-Solution. *Anal. Chem.* **1995**, *67*, 3391–3400.
2. Zhang, H.; Davison, W.; Knight, B.; McGrath, S. In situ measurements of solution concentrations and fluxes of trace metals in soils using DGT. *Environ. Sci. Technol.* **1998**, *32*, 704–710.
